# Fabrication of Loose Nanofiltration Membranes with High Rejection Selectivity between Natural Organic Matter and Salts for Drinking Water Treatment

**DOI:** 10.3390/membranes12090887

**Published:** 2022-09-15

**Authors:** Zhihai He, Kunpeng Wang, Yanling Liu, Ting Zhang, Xiaomao Wang

**Affiliations:** 1 State Key Joint Laboratory of Environment Simulation and Pollution Control, School of Environment, Tsinghua University, Beijing 100084, China; 2 State Key Laboratory of Pollution Control and Resource Reuse, College of Environmental Science and Engineering, Tongji University, Shanghai 200092, China; 3 School of Environmental Science and Engineering, Tianjin University, Tianjin 300350, China

**Keywords:** loose nanofiltration (LNF), non-solvent induced phase separation (NIPS), interfacial polymerization (IP), water treatment, natural organic matter (NOM), rejection selectivity

## Abstract

Loose nanofiltration (LNF) membranes with a molecular weight cut-off (MWCO) of about 1000 Da and high surface negative charge density have great application potential for drinking water treatment pursuing high rejection selectivity between natural organic matter (NOM) and mineral salts. This study was conducted to exploit the novel method coupling non-solvent induced phase separation (NIPS) and interfacial polymerization (IP) for the preparation of high-performance LNF membranes. A number of LNF membranes were synthesized by varying the polyethersulfone (PES) and piperazine (PIP) concentrations in the cast solution for the PES support layer preparation. Results showed that these two conditions could greatly affect the membrane water permeance, MWCO and surface charge. One LNF membrane, with a water permeance as high as 23.0 ± 1.8 L/m^2^/h/bar, when used for the filtration of conventional process-treated natural water, demonstrated a rejection of NOM higher than 70% and a low rejection of mineral salts at about 20%. Both the mineral salts/NOM selectivity and permselectivity were superior to the currently available LNF membranes as far as the authors know. This study demonstrated the great advantage of the NIPS–IP method for the fabrication of LNF membranes, particularly for the advanced treatment of drinking water.

## 1. Introduction

Nanofiltration (NF) membranes, with a molecular weight cut-off (MWCO) in the range from 200 to 2000 Da, have a great application potential for the advanced treatment of drinking water. Nevertheless, the MWCO of most commercial NF membranes currently used for water treatment is generally within 200–500 Da, which ensures the high performance in removing not only natural organic matter (NOM) but also trace organic compounds (TrOCs) [1,2]. Accordingly, the water permeances of these membranes are relatively low, hardly higher than 15 L/m^2^/h/bar. In addition, the use of these membranes could remove excessive mineral salts including hardness (calcium/magnesium) ions from water, usually higher than 50%, resulting in a decrease in not only water chemical stability but also the water recovery rate [3,4,5]. Moreover, low contents of hardness ions and total dissolved solids (TDS) in drinking water would impair its taste [6] and even healthiness. Indeed, the World Health Organization (WHO) recommends a minimum TDS of 100 mg/L for drinking water [7].

The use of loose NF (LNF) membranes could be an alternative option for drinking water treatment, as these are capable of highly effectively removing NOM while maintaining most of the mineral salts in the treated water [8]. LNF membranes are defined as having an MWCO higher than 500 Da [9], and are more suitable for drinking water treatment only if the MWCO is about 1000 Da and carrying sufficiently high surface negative charges. It is noteworthy that about 90% of NOM have a molecular weight in the range of 500–3000 Da, and, as with conventional NF membranes, LNF membranes rely on steric exclusion and an electrostatic effect for the rejection of charged solutes [10,11]. Most of the TrOCs remaining in the LNF-treated water, which have relatively low molecular weights, if any, could be more cost-effectively removed by a subsequent physical adsorption or chemical oxidation, owing to the low content of co-existing NOM. This combined process could be economically viable due to the enhanced membrane water permeance (and thus lowered membrane area and energy consumption), increased water recovery rate and greatly reduced requirement of post-treatment such as the supplementation of essential mineral ions (re-mineralization) and pH adjustment [12]. Additionally, LNF membranes could have many other applications relating to material separation, in addition to drinking water treatment [9,13,14,15].

Currently, the main methods for LNF membrane synthesis include the classical interfacial polymerization (IP), phase separation, polyphenol/polyamine deposition and surface coating, among which the first two are the most commonly used [16,17]. In this study, the novel method coupling non-solvent induced phase separation (NIPS) and IP was applied for LNF membrane preparation, targeting an MWCO of about 1000 Da. The key feature of the NIPS–IP method is that the aqueous monomer for IP is preloaded into the matrix of porous support prepared by NIPS. The NIPS–IP method, using piperazine (PIP) and trimesoyl chloride (TMC) as the aqueous and organic-phase monomers, respectively, was reported in our previous study for the synthesis of conventional NF membranes targeting a higher water permeance [18]. This work is an extension of the application of the method, which, in synthesizing LNF membranes, would be advantageous over the phase separation method since it could effectively reduce the formation of defects including too large membrane pores. In this study, both the NIPS and IP conditions were optimized so as to regulate the MWCO of LNF membranes. The performance of the prepared LNF membrane was tested by filtering synthetic and natural water and compared with representative commercially available LNF membranes.

## 2. Materials and Methods

### 2.1. Synthesis of LNF Membranes

**Figure 1:**
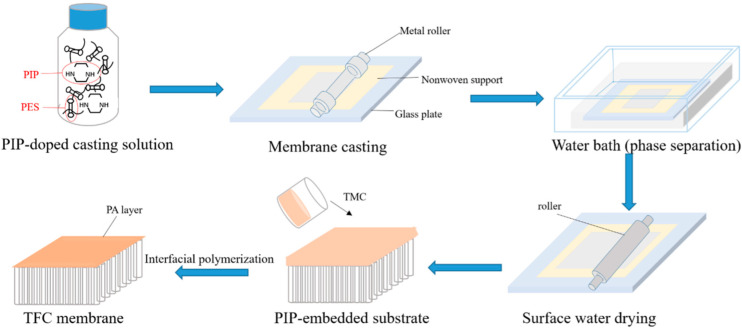
Schematic procedure of preparing LNF membranes by the NIPS–IP method.

Figure 1 schematically shows the procedure of preparing LNF membranes by adopting the NIPS–IP method. In brief, the casting solution was prepared by dissolving an amount of PIP monomer (99% purity, Sigma-Aldrich, St. Louis, MO, USA) in a volume of DMSO (99.5% purity, ThermoFisher, Waltham, MA, USA), followed by adding an amount of polyethersulfone (PES) powder (Veradel 3000P) under a continuous stirring condition with a magnetic stirrer for 12 h until the solute was completely dissolved. After degasification, the viscous bubble-free solution was uniformly applied, using a metal roller, to the surface of a non-woven fabric fixed on a glass plate, with a thickness of 200 μm, which was immediately submerged into 0.7 L of deionized (DI) water (25 °C). After a duration of 15 s for phase separation in the water bath, the solidified support was removed from the water bath, and the water droplets on the support layer surface, if any, were removed with a rubber roller. Thereafter, 50 mL n-hexane (>98.5% purity, ThermoFisher) solution of TMC (98% purity, Sigma-Aldrich) with a weight concentration of 0.1% TMC was poured onto the surface of the PIP-loaded support having a surface area of 15 × 15 cm^2^, and the IP reaction was allowed to take place for 1 min. After removing the excess liquid, the synthesized thin-film composite (TFC) membrane was dried at room temperature (~25 °C) for 5 min. The membrane was then washed several times with DI water to remove any residual chemicals and stored in DI water (4 °C) before testing.

A total of six TFC membranes were synthesized by varying the PES and PIP weight concentrations in the casting solution in the ranges of 13–17% and 0.4–1.2%, respectively (Table 1). At least two replicates were prepared for each membrane. The membranes were characterized for the surface and cross-section morphologies by using atomic force microscopy (AFM, Dimension ICON, Bruker, Germany) and field emission scanning electron microscopy (FESEM, Hitachi S5500, Japan).

### 2.2. Test of Membrane Performance

The synthesized membranes were tested for the water permeance and MWCO as well as the rejection of mineral salts and NOM by using a lab-scale cross-flow NF system with three parallel cells (CF016D, Sterlitech, Auburn, WA, USA) as shown in Figure S1. Each filtration cell had an effective area of 20.6 cm^2^. During all filtrations, the water temperature was maintained at 23 °C by using a thermostat. The three membrane coupons of one membrane batch were compacted at 6 bar for about 1 h until the water flux was stable, before the applied pressure was adjusted to 4 bar for filtration. The water flux was measured for the calculation of membrane water permeance. Polyethylene glycol (PEG) solutions and β-cyclodextrin (MW = 1143 Da, 98%, Sigma-Aldrich) with a concentration of 0.1 g/L were filtered for the determination of MWCO of membrane. Five PEGs were used, with a mean molecular weight of 200, 300, 600, 1000 and 2000 Da (obtained from Sinopharm Chemical Reagent Co., Ltd., Sigma-Aldrich, or Aladdin), respectively. The concentration of mineral salt, being Na_2_SO_4_, NaCl or MgCl_2_, was 1.0 g/L, which was filtered to estimate the membrane surface charge density. The (apparent) rejection of a solute is determined from the solute concentrations in the feed water (c_f_) and in the permeate (c_p_).

R = (1 − c_p_/c_f_) × 100% (1)

The solute permeability coefficient (B value) could be calculated by

B = J_w_ (1/R − 1) × 100% (2)

The water flux (J_w_) in (2) is calculated from the relationship between the membrane water permeance (A value) and applied pressure (ΔP)

J_w_ = A ΔP (3)

To determine maximum pressure and compressibility of prepared membranes, LNF2 was selected for testing. By taking Na_2_SO_4_ solution as feed water, three coupons of LNF2 membrane were compacted at 6 bar for about 2 h, before the applied pressure was adjusted from 2 to 12 bar for filtration. After lasting for 2 h at each pressure, the water flux and Na_2_SO_4_ rejection were tested.

According to NF theories, an LNF membrane with high surface negative charge density would demonstrate a characteristic rejection order of Na_2_SO_4_ > NaCl > MgCl_2_ [10]. To test the rejection of NOM, particularly by LNF2, one synthetic solution of fulvic acid (Macklin Biochemical, Shanghai, China) at a concentration of 2.3 mg/L as total organic carbon (TOC) and two treated natural waters were used. One water was collected from the carbon filter effluent of a pilot-scale water treatment plant in Beijing, which had a TOC content of 3.0 mg/L, and the other from the effluent of a high-rate settling tank at a full-scale water treatment plant in Shandong Province, China, which had a TOC content of 2.5 mg/L. The raw waters were surface water for both plants. 

During filtration, the feed and permeate water might be sampled for the determination of the conductivity and TOC content, by a conductivity meter (Shimadzu, China) and a TOC analyzer (TOC-V_CPH_, Shimadzu, China), respectively, so as to calculate the respective rejection. 

In addition, some of the water samples were determined for the fluorescence excitation–emission (FEEM) spectra by using a fluorescence spectrometer (F-7000, Hitachi). The obtained FEEM spectra were corrected and standardized following the procedure that was reported previously [19]. According to the literature [20], the as-obtained FEEM spectrogram was divided into five regions, each representing a specific fraction of DOM. Specifically, Region I (E_x_/E_m_ = (200–250)/(250–330) nm) and Region II (E_x_/E_m_ = (200–250)/(330–380) nm) were assigned to tryptophan-like and tyrosine-like proteins, respectively, and Region III (E_x_/E_m_ = (200–250)/(380–600) nm), Region IV (E_x_/E_m_ = (250–400)/(250–380) nm) and Region V (E_x_/E_m_ = (250–500)/(380–600) nm) were assigned to fulvic acid-like, soluble microbial product (SMP)-like and humic acid-like substances, respectively. 

## 3. Results and Discussion

### 3.1. Effect of PES Concentration

**Figure 2:**
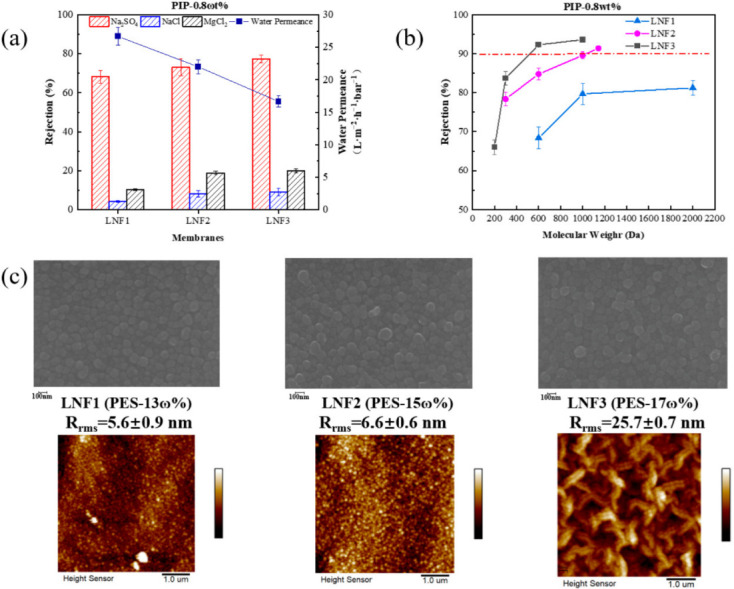
Effect of PES concentration on the performance and properties of the TFC LNF membranes. The PIP concentration in the cast solution was fixed at 0.8%. (**a**) Water permeance and rejections of Na_2_SO_4_, NaCl and MgCl_2_. (**b**) Rejections of PEGs with different molecular weight. (**c**) Membrane surface morphology depicted by SEM and AFM images.

Based on the preliminary exploration, the weight percentage of PIP in the cast solution for the support layer preparation was fixed at 0.8%, and the weight percentage of PES was changed from 13% to 17%. The membrane water permeance, rejections of different single salts and rejections of different PEGs were determined for the three TFC membranes (Figure 2). Results showed that when the concentration of PES increased, the membrane water permeance decreased substantially from 27 L/m^2^/h/bar (for LNF1) to 17 L/m^2^/h/bar (for LNF3), while the rejection of each single salt increased slightly (Figure 2a) and, as such, the B value of each single salt decreased (Table S1). From the membrane rejections of PEG with different molecular weights (Figure 2b), it can be inferred that the membrane MWCO decreased when the PES concentration increased. This was because, with the increase in PES concentration, the viscosity of the casting solution increased so that the diffusion resistance of PIP also increased. As a result, the concentration of PIP remaining on the surface of the support layer formed after phase separation was higher, which led to a smaller pore size of the fabricated NF membrane. For each membrane, the rejection of Na_2_SO_4_ (68–77%) was much higher than that of MgCl_2_ (10–20%), which was nevertheless higher than that of NaCl (4–9%). This infers that the membranes fabricated under these conditions were negatively charged on the surface but not to a very high level [21,22]. 

It is worth noting that the rejections of PEG-1000 and PEG-2000 by LNF1, and some membranes described later, were quite similar. The main reason was that the PEG molecules have a chain (linear) structure, which would be rejected by a lower value when compared with spherical molecules with the same molecular weight [23,24,25]. It might infer that PEGs, especially those with relatively large molecular weights, may not be suitable for the determination of the membrane MWCO. The LNF2 membrane, fabricated with a PES concentration of 15% and a PIP concentration of 0.8%, had a membrane water permeance of 23 L/m^2^/h/bar, a rejection of PEG-1000 at ~89% and a rejection of β-cyclodextrin at ~92%. The MWCO of this membrane (LNF2) was estimated to be 1050 Da (Table 1).

The SEM images of the membrane top surface showed that all membranes were relatively flat, typical of semi-aromatic NF membranes, though the AFM images showed that the membrane surface roughness increased to a relatively high level of 25.7 nm when the PES weight percentage increased to 17%. Leaf-like structures, typical of reverse osmosis membranes, could be observed on the membrane surface. This indicates that when the PES concentration was higher, the amount of PIP remaining on the support layer surface was higher, and the subsequent IP reaction would be more intense and produce more rough structures [26]. An increased roughness could substantially affect the membrane fouling behavior when in use, resulting from the change in the hydrodynamic characteristic in the vicinity of the membrane surface [27,28]. In addition, the appearance of protuberances could increase the effective area of water permeation. Nevertheless, the membrane water permeance was lowest when the PES concentration was 17%, which was due to the densest polyamide active layer formed by IP.

### 3.2. Effect of PIP Concentration

**Figure 3:**
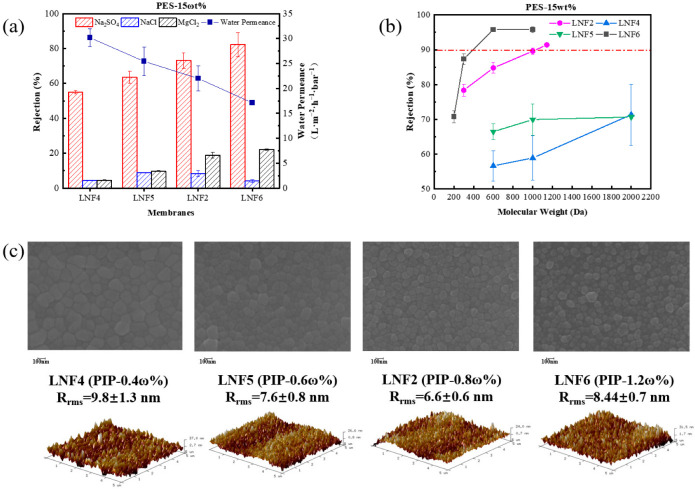
Effect of PIP concentration in the cast solution on the performance and properties of the TFC LNF membranes. The PES concentration in the cast solution was fixed at 15%. (**a**) Water permeance and rejections of Na_2_SO_4_, NaCl and MgCl_2_. (**b**) Rejections of PEGs with different molecular weights. (**c**) Membrane surface morphology depicted by SEM and AFM images.

When the PES concentration in the cast solution was fixed at 15%, and the PIP concentration increased from 0.4% to 1.2%, the membrane water permeance, rejection ability and B value of each single salt changed greatly (Figure 3 and Table S1). As the PIP concentration increased, the membrane water permeance decreased from 30 L/m^2^/h/bar (for LNF4) to 17 L/m^2^/h/bar (for LNF6). This was again mainly because of the increasingly dense active layer structure and reduced membrane pore size, illustrated by the greatly increased rejection of PEGs, especially when the PIP concentration was increased to 0.8% or higher. As such, the membrane’s ability of rejecting Na_2_SO_4_ greatly increased from 55% (for LNF4) to 82% (for LNF6), and the rejection of MgCl_2_ also increased substantially. The much higher rejection of Na_2_SO_4_ than MgCl_2_ by each membrane indicates that all membranes were negatively charged on the surface. Nevertheless, the first increase and then decrease in the rejection of NaCl with the increase in PIP concentration indicates that the membrane surface became less negatively charged, in consideration of the increase in membrane pore size, which was favorable for solute rejection by the steric exclusion effect. 

It is noteworthy that when the PIP concentration was relatively low, rejections of PEGs differed substantially among the membrane replicates fabricated under the same condition, illustrated by the large error bars. This indicates that the NIPS–IP method might not be sufficiently reproducible when the PIP concentration is low and the pore sizes of the fabricated membrane could be highly non-uniform [29]. This could be due to the non-uniform distribution of PIP monomers in the PES support layer and the deficiency of PIP monomers in some areas to react with TMC monomers during the IP step, resulting in large and massive defects in the fabricated membrane.

The AFM and SEM characterizations showed that as the PIP concentration increased, the membrane surface roughness did not change substantially (Figure 3c), not as much as it did when the PES concentration increased. The relatively high roughness (9.8 nm) when the PIP concentration was low at 0.4% was probably due to the many defects generated while the relatively high roughness (8.4 nm) for the membrane prepared with a PIP concentration of 1.2% was a result of the massive formation of nodules or protrusions on the surface. Nevertheless, all these membranes had a level of roughness value typical of semi-aromatic NF membranes.

**Figure 4:**
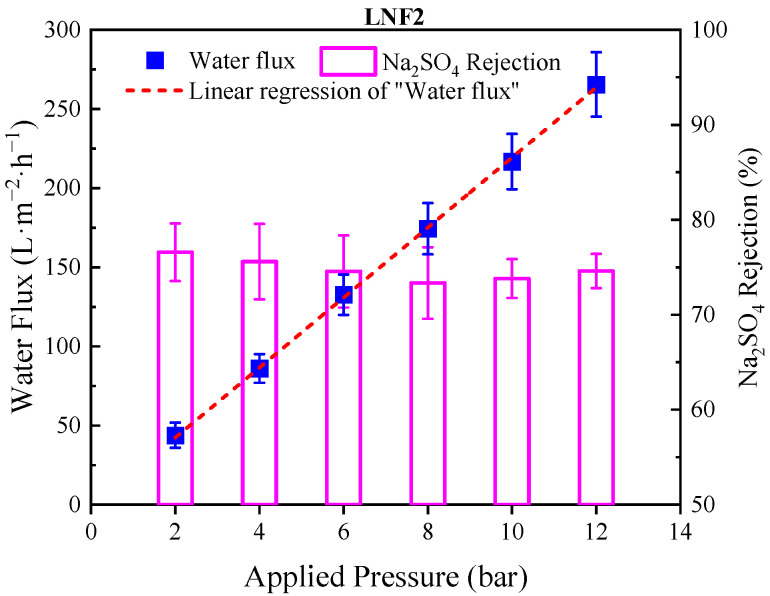
Water flux and Na_2_SO_4_ rejection for LNF2 membrane tested at different pressures. The straight red dashed line is linear regression of water flux-applied pressure relationship with the slope (k) equal to 22.5 L/m^2^/h/bar, which was identical to the A value of membrane.

It is noteworthy that the membranes prepared by the NIPS–IP method could endure a high applied pressure with very low compressibility. Taking the LNF2 membrane as an example, which had an MWCO of 1050 Da, by increasing the applied pressure from 2 to 12 bar, the performance of the LNF2 membrane in rejecting Na_2_SO_4_ was stable, and the membrane water flux increased linearly with the applied pressure (Figure 4).

### 3.3. Rejection Selectivity of the Prepared Membrane

Our previous study [10] showed that LNF membranes with an MWCO of ~1000 Da and sufficiently high surface negative charge density could have a high mineral salts/NOM selectivity, with rejections of NOM and mineral salts higher than 70% and lower than 30%, respectively. However, the water permeance of currently available LNF membranes need to be further improved. Table 2 shows some of the commercially available and lab-made LNF membranes, with MWCO all higher 600 Da. It is clear that the LNF2 membrane had a higher water permeance than those commercial LNF membranes listed with a similar MWCO.

**Figure 5:**
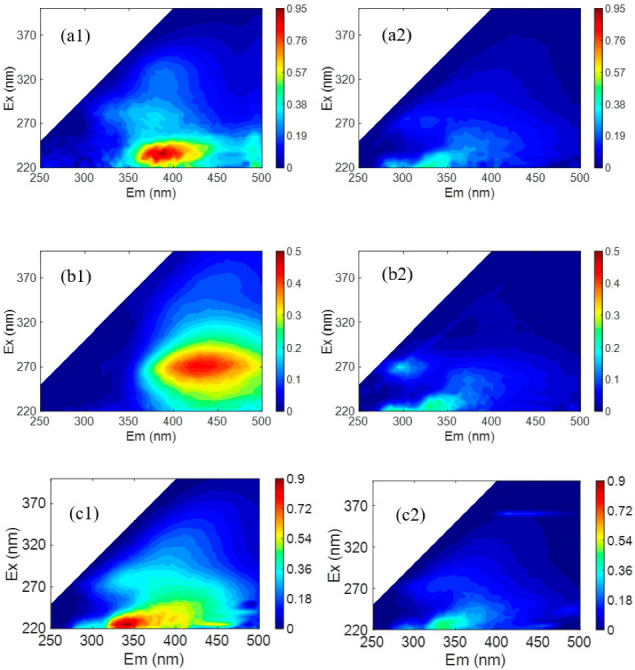
FEEM spectra of the feed water (on the left) and the permeate water (on the right) when LNF2 was used. (**a1**) An activated carbon filter effluent collected from a local pilot plant and (**a2**) the permeate; (**b1**) the synthetic feed water containing fulvic acid and (**b2**) the permeate; (**c1**) the effluent of a high-rate settling tank at a surface water treatment plant in Shandong and (**c2**) the permeate.

**Figure 6:**
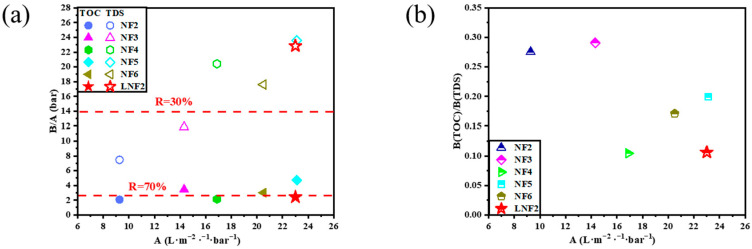
Comparison of LNF2 with five commercial LNF membranes by plotting the dependence of (**a**) the inverse water/solute (i.e., NOM and TDS) selectivity and (**b**) the NOM/mineral salts selectivity on the membrane water permeance.

To test the mineral salts/NOM selectivity of the LNF2 membrane prepared by the novel NIPS–IP method, two treated natural surface waters were used. The first one was the effluent of an activated carbon filter of a pilot-scale treatment plant in Beijing, which had a TOC of 3.0 mg/L and a TDS of 197.8 mg/L. At an applied pressure of 4 bar, the LNF2 membrane could reject NOM as TOC by 70.8%, while allowing most of the mineral salts to pass through with a low TDS rejection of 19.0%. These results showed that the LNF2 membrane had a high rejection selectivity between NOM and mineral salts. A comparison of the FEEM spectra of the feed water and the permeate water (Figure 5(a1,a2)) showed that the fulvic acid-like substances (Region III) contained in the raw water were largely removed, while tyrosine-like proteins (Region II), though having a low concentration in the feed water, mostly remained in the permeate water. As with our previous study [42], this result showed that the steric hindrance and electrostatic effects were both responsible for the rejection of NOM. Fulvic acid-like substances are among the NOM components that have a relatively low molecular weight and are, thus, expected to have relatively low rejection. To test this hypothesis, a synthetic feed water containing commercial fulvic acids as the only group of organic solutes was filtered by using the LNF2 membrane. The synthetic water had a TOC content of 2.3 mg/L and a TDS of 198.7 mg/L. Indeed, the FEEM spectra of feed water (Figure 5(b1)) showed a strong excitation–emission peak characteristic of fulvic acid in Region III. A comparison with the FEEM for the permeate water (Figure 5(b2)) showed that most of the fulvic acid was removed by the LNF2 membrane. However, measurement of the TOC content in the permeate water (0.94 mg/L) showed that the NOM rejection was 59%. (In comparison, the rejection of mineral salts as TDS was 15.7%.) This indicates that a substantial portion of fulvic acids, most of which were non-fluorescent, could not be well rejected by LNF membranes such as LNF2, which either had a low molecular weight or carried little molecular charge, and thus were not favorable to being rejected by the steric hindrance and electrostatic effect, respectively. The content of these non-fluorescent fulvic acids in natural waters need to be further studied. To compare the mineral salts/NOM selectivity of LNF2 with the commercial LNF membranes, a second treated natural water was used, which was collected from a high-rate settling tank at a large-scale surface water treatment plant in Shandong Province. The water had a TOC of 2.5 mg/L and a TDS of 272.1 mg/L. The FEEM spectra showed that the feed water had more tyrosine-like proteins and less fulvic acid-like substances than the first feed water. A comparison of the FEEM spectra of the feed and permeate water (Figure 5(c1,c2)) again showed that most of the (fluorescent) fulvic acid-like substances were removed while a substantial proportion of the tyrosine-like proteins remained in the permeate water. Nevertheless, the NOM (as TOC) rejection by the LNF2 membrane was still high at 71.2%, along with a low rejection of mineral salts (as TDS) at 20.8%. The mineral salts/NOM selectivity of LNF2 was compared with that of five commercial LNF membranes (NF2–NF6 in Table 2), which were tested by using the same feed water as reported in Zhang et al. [10] (Figure 6). It is clear that the LNF2 membrane had the highest mineral salts/NOM selectivity among the six LNF membranes, along with a high membrane water permeance of about 23.0 L/m^2^/h/bar. The better performance of LNF2 regarding selectivity was likely owing to the higher membrane surface negative charge density, given that the MWCO was similar or even higher than commercial membranes. This clearly demonstrated the high application potential of the NIPS–IP method in fabricating high-performance LNF membranes pursuing high mineral salts/NOM selectivity for the advanced treatment of drinking water.

## 4. Conclusions

This study clearly demonstrated that the novel NIPS–IP method could be extended for the fabrication of LNF membranes for the advanced treatment of drinking water. It was found that both PIP and PES concentrations in the cast solution for the PES support layer fabrication could greatly affect the membrane MWCO and surface charge, and, in turn, the water permeance and rejection characteristics of mineral salts. Lower PIP and PES concentrations could increase the membrane MWCO but might also cause the formation of more defects. To fabricate LNF membranes suitable for water treatment, one set of optimum conditions were as follows: the weight percentages of PIP and PES are 0.8% and 15% in the cast solution in DMSO, respectively, the duration of phase separation time is 15 s, the weight percentage of TMC is 0.1% in n-hexane and the time of IP is 1 min. The prepared LNF membrane had an MWCO of about 1000 Da and relatively abundant negative charges on the surface. Filtration tests using one synthetic solution of fulvic acid and two treated natural waters confirmed that the membrane had the characteristics of high water permeance (>20 L/m^2^/h/bar), high NOM rejection (>70%) and low TDS rejection (<20%), all favorable for drinking water treatment. Both the mineral salts/NOM selectivity and permselectivity were superior to the currently available LNF membranes tested in our previous studies. The NIPS–IP method could be applied for the fabrication of LNF membranes for other valuable applications.

## Figures and Tables

**Table 1:** The preparation conditions and characteristics of the six LNF membranes.

Membrane	PES Concentration	PIP Concentration	TMC Concentration	MWCO (Da)	Water Permeance (L/m 2/h/bar)
LNF1	13 wt%	0.8 wt%	0.1 wt%	>2000	26.7 ± 1.3
LNF2	15 wt%	0.8 wt%	0.1 wt%	1050	23.0 ± 1.1
LNF3	17 wt%	0.8 wt%	0.1 wt%	530	16.7 ± 0.8
LNF4	15 wt%	0.4 wt%	0.1 wt%	>2000	30.2 ± 1.8
LNF5	15 wt%	0.6 wt%	0.1 wt%	>2000	25.4 ± 2.9
LNF6	15 wt%	1.2 wt%	0.1 wt%	400	17.1 ± 0.1

**Table 2:** Main information of commercially available LNF membranes.

Membrane Code	Supplier	Membrane Material	MWCO (Da)	Water Permeance (L/m2/h/bar)	References
CK	GE Osmonics	Cellulose acetate	~2000	3.45	[30]
GE	GE Osmonics	Polyamide	1000	1.11	[30]
GH	GE Osmonics	Polyamide	2000	3.29	[30]
GK	GE Osmonics	Polyamide	2000	10.0	[30]
NP010	Microdyn Nadir	Polyether sulfone	~1000	>5.0	[30]
TriSep SBNF	Microdyn Nadir	Cellulose acetate	2000	12.0~17.7	[30]
NFPES10	Microdyn Nadir	Polyether sulfone	1000	15.4	[30]
Sepro NF6	Ultura	Polyamide	850	16.7	[30]
NF2 *	–	Polyamide	610	9.27	[10]
NF3 *	–	Polyamide	660	14.3	[10]
NF4 *	TriSep	Polyamide	970	16.9	[10]
NF5 *	Synder	Polyamide	1050	23.1	[10]
NF6 *	–	Polyamide	1240	20.5	[10]
–	Lab-made	PES	1250	20.0	[31]
–	Lab-made	PES with sulfonated halloysite nanotubes	706	18.7	[32]
–	Lab-made	Diethylenetriamine-TMC	800	4.5	[33]
–	Lab-made	Sericin-TMC	880	11.9	[34]
–	Lab-made	Polyethyleneimine-Trimesic acid	1000	19.1	[35]
–	Lab-made	Triethanolamine-TMC	1490	8.6	[36]
–	Lab-made	(NH2-PEG600)- TMC	678	13.2	[37]
–	Lab-made	Chitin xanthate/H_2_O_2_	652	2.8~3.8	[38]
–	Lab-made	Gallic acid-polyethyleneimine	950	18.0	[39]
–	Lab-made	Polydopamine	1250	17.5	[40]
–	Lab-made	Hybrid of poly(1,4-phenylene ether ether sulfone), polyacrylonitrile, poly(vinyl pyrrolidone), and SBA-15 mesoporous silica	1000	1.3~13.3	[41]

Note: The LNF membranes with a * mark were tested in our previous study [10] for the mineral salts/NOM selectivity by using treated natural water as well as MWCO and water permeance, for comparative purpose.

## Data Availability

Not applicable.

## Supplementary Materials

The following supporting information can be downloaded at: https://www.mdpi.com/article/10.3390/membranes12090887/s1, Figure S1: Schematic diagram of the cross-flow nanofiltration setup comprising of three parallel filtration cells; Table S1: The B values of prepared membranes for monovalent and divalent ions.
