# Supplementary material for: Fabrication of Loose Nanofiltration Membranes with High Rejection Selectivity between Natural Organic Matter and Salts for Drinking Water Treatment
Source: Membranes (Basel). 2022 Sep 15;12(9):887. doi: 10.3390/membranes12090887 (PMC9501612; doi:10.3390/membranes12090887)
Supplement: Supplementary file 1 [file membranes-12-00887-s001.zip › membranes-1883237-supplementary.pdf]

Supporting Information

Fabrication of Loose Nanofiltration Membranes with High Rejection Selectivity between Natural Organic Matter and Salts for Drinking Water Treatment

Zhihai He [1], Kunpeng Wang [1], Yanling Liu [2], Ting Zhang [3] and Xiaomao Wang [1,*]

[1] State Key Joint Laboratory of Environment Simulation and Pollution Control, School of Environment, Tsinghua University, Beijing 100084, China

[2] State Key Laboratory of Pollution Control and Resource Reuse, College of Environmental Science and Engineering, Tongji University, Shanghai 200092, China

[3] School of Environmental Science and Engineering, Tianjin University, Tianjin 300350, China;

* Correspondence: wangxiaomao@tsinghua.edu.cn; Tel.: +86-10-6278-1386

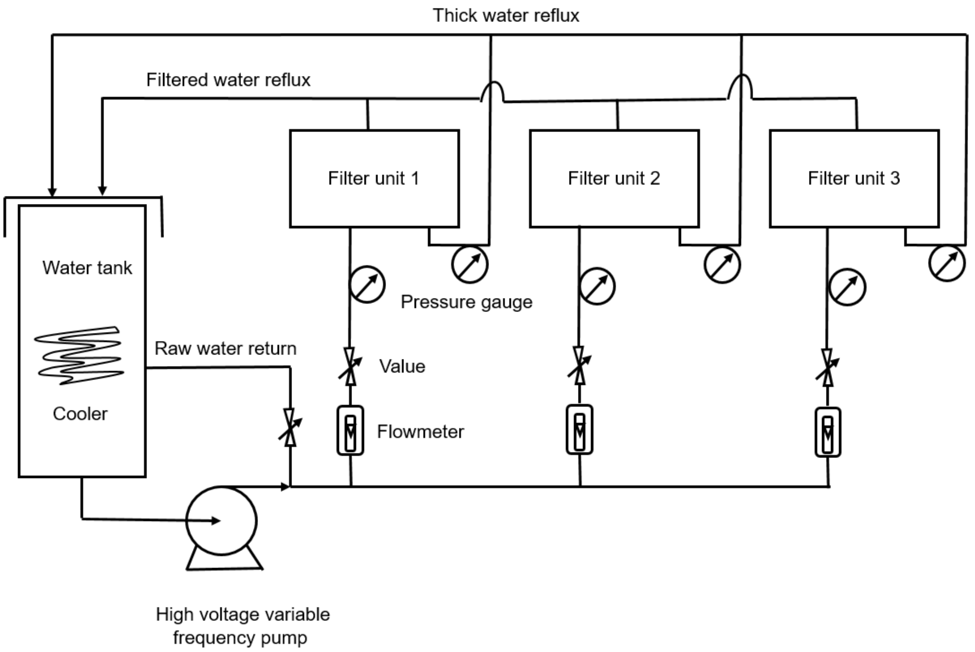

Figure S1. Schematic diagram of the cross-flow nanofiltration setup comprising of three parallel filtration cells.

Table S1. The B values of prepared membranes for monovalent and divalent ions.

Membrane	B (Na_2SO_4) (L/m2/h)	B (NaCl) (L/m2/h)	B ($MgCl_2$) (L/m2/h)
LNF1	49.8	2330.1	927.5
LNF2	33.9	1013.8	394.8
LNF3	19.5	676.8	266.1
LNF4	98.9	2682.3	2557.8
LNF5	58.3	1031.4	941.9
LNF6	14.8	1639.5	239.6
